# Fried foods: a risk factor for laryngeal cancer?

**DOI:** 10.1038/sj.bjc.6600639

**Published:** 2002-11-12

**Authors:** C Bosetti, R Talamini, F Levi, E Negri, S Franceschi, L Airoldi, C La Vecchia

**Affiliations:** Istituto di Ricerche Farmacologiche ‘Mario Negri’, Via Eritrea 62, 20157 Milan, Italy; Servizio di Epidemiologia, Centro di Riferimento Oncologico, Via Pedemontana Occ.le, 33081 Aviano (Pordenone), Italy; Registre vaudois des tumeurs, Institut universitaire de médicine sociale et préventive, CHUV-Falaises 1, 1011 Lausanne, Switzerland; International Agency for Research on Cancer, 150 Cours Albert Thomas, F-69372 Lyon, cédex 08, France; Istituto di Statistica Medica e Biometria, Università degli Studi di Milano, Via Venezian 1, 20133 Milan, Italy

**Keywords:** laryngeal cancer, diet, fried foods, risk factors, case–control study

## Abstract

The role of fried foods on laryngeal cancer risk was investigated in a case–control study from Italy and Switzerland on 527 cases and 1297 hospital controls. A significant increased risk was found for high consumption of fried meat, fish, eggs and potatoes, with odds ratios of 1.6, 3.1, 1.9 and 1.9, respectively.

*British Journal of Cancer* (2002) **87**, 1230–1233. doi:10.1038/sj.bjc.6600639 www.bjcancer.com

© 2002 Cancer Research UK

## 

The relation between cooking methods such as frying, boiling and barbecuing and cancer risk has been inadequately investigated in epidemiological studies (Archer, 1988; Steineck *et al*, 1993). Among the few studies considering this issue, some investigations reported an increased risk of cancer of the colon, rectum and stomach in relation to consumption of fried foods (mostly meat) (IARC, 1993; Steineck *et al*, 1993), and a few others found a positive association with other common non digestive neoplasms, including breast (Phillips, 1975; Knekt *et al*, 1994; de Stefani *et al*, 1997a), oesophageal (Ward *et al*, 1997), lung (Sinha *et al*, 1998a), pancreatic cancer (Norell *et al*, 1986) and low urinary tract (Steineck *et al*, 1990).

During the process of frying protein-rich foods, such as meat and fish, various kinds of mutagenic and carcinogenic heterocyclic amines (HA) are produced, particularly when cooking temperature is very high (Övervik and Gustafsson, 1990; Layton *et al*, 1995; Skog *et al*, 1998). The fats used for frying seem to further increase the mutagenic activity of HA, probably enhancing heat conduction (Övervik and Gustafsson, 1990; Skog *et al*, 1998). Heterocyclic amines have been shown to cause malignant tumours in the colon and breast of mice and rats, and are possible or probable carcinogens for humans (IARC, 1993). Fried foods other than meat or fish contain only small amounts of HA and have therefore low mutagenic activity (Bjeldandes *et al*, 1982a,b). However, some epidemiological studies have reported an increased risk of cancer with consumption of fried eggs and potatoes as well (Phillips, 1975; Demirer *et al*, 1990; Steineck *et al*, 1990).

The larynx is directly exposed to volatile carcinogens in tobacco smoking. Besides tobacco and alcohol consumption, which are the main recognised risk factors, diet has been associated with laryngeal cancer risk (World Cancer Research Fund, 1997). However, scanty data exists on the potential role of carcinogens present in fried meat or other foods with specific reference to laryngeal cancer. A case–control study in China on 201 incident cases and 414 controls, reported that consuming deep-fried foods ‘daily’, as opposed to ‘never/occasionally’, increased the risk of laryngeal cancer (odds ratio (OR)=2.5, 95% confidence interval (CI)=1.0–6.1), although no clear dose-response relation was observed (Zheng *et al*, 1992). A case–control study in Uruguay on cancers of the upper aerodigestive tract found no association with consumption of fried meat, but reported an increased risk in relation to total HA intake whose effect was similar for different cancer sites (de Stefani *et al*, 1997b).

We have therefore investigated the possible relation between fried food intake and laryngeal cancer risk using data from a large case–control study conducted in Italy and Switzerland, which reported a protective effect of vegetables, fruit, and olive oil and a detrimental effect of meat and other protein rich foods (Bosetti *et al*, 2002).

## MATERIALS AND METHODS

The present study is based on data of a case–control study of laryngeal cancer conducted between 1992 and 2000 in two Italian areas (the provinces of Pordenone and the greater Milan area) and in the Swiss Canton of Vaud (Bosetti *et al*, 2002). Cases were 527 patients (478 men and 49 women, median age 61 years, range 30–79) admitted to teaching and general hospitals in the areas under study with incident, histologically confirmed squamous-cell carcinoma of the larynx, diagnosed no longer than 1 year before the interview. These included 271 glottis, 117 supraglottis, 4 subglottis and 135 other or unspecified laryngeal cancers. Controls were 1297 subjects (1052 men and 245 women, median age 61 years, range 31–79) selected among patients admitted to the same hospitals for a wide spectrum of acute, non-neoplastic conditions, not related to smoking, alcohol consumption and long-term modification of diet. These were frequency-matched to cases by 5-year groups, sex and area of residence; to compensate for the rarity of laryngeal cancer in women, a control-to-case ratio of about five was chosen for females, as apposed to two for males. Among the controls, 27% were admitted for traumas, 22% for other orthopaedic disorders, 29% for acute surgical conditions, and 23% for miscellaneous other illnesses, including eye, nose, ear, skin or dental disorders.

Cases and controls were interviewed during their hospital stay using a structured questionnaire, including information on socio-demographic characteristics, anthropometric measures, life-style habits, such as tobacco smoking and alcohol drinking, a personal medical history and family history of cancer in first-degree relatives.

The subjects' usual diet during the 2 years prior to cancer diagnosis or hospital admission (for controls) was investigated through an interview-administered food freqeuncy questionnaire (FFQ), previously tested for validity (Decarli *et al*, 1996) and reproducibility (Franceschi *et al*, 1993). The FFQ included 78 foods and beverages, as well as a range of recipes, grouped into seven sections: (i) bread and cereal dishes (first courses); (ii) meat and other main dishes (second courses); (iii) vegetables (side dishes); (iv) fruit; (v) sweets, desserts and soft drinks; (vi) milk, hot beverages and sweeteners; (vii) alcoholic beverages. Specific questions referred to fried foods, such as beef or veal, fish or shellfish, eggs or omelette and potatoes. No information was available on how long the meat was cooked. Subjects were asked to indicate the average weekly frequency of consumption of each dietary item; intakes lower than once a week, but at least once a month, were coded as 0.5 per week. To estimate total energy intake an Italian food composition database was used (Salvini *et al*, 1996). Further questions aimed to assess the fat intake pattern were also included in the questionnaire, and used to derive quantitative estimates of intake of various seasoning fats.

Odds ratios (OR) and the corresponding 95% confidence intervals (CI) for each category of intake of fried foods (low, medium, high) were estimated using unconditional multiple logistic regression models (Breslow and Day, 1980). All models were adjusted for age, sex, area of residence, years of education, tobacco smoking, alcohol drinking, and non-alcohol energy intake (Willett and Stampfer, 1986). Tests for trend were based on the likelihood ratio test between models with and without a linear term for each food group.

## RESULTS

Table 1

**Table 1:**
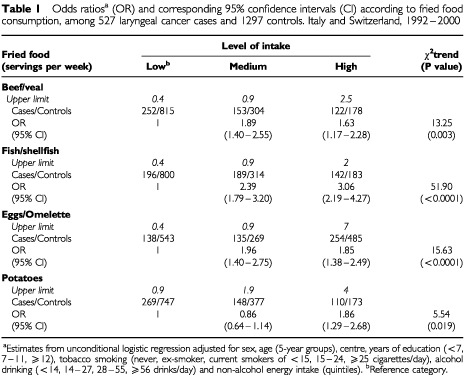
Odds ratios^a^ (OR) and corresponding 95% confidence intervals (CI) according to fried food consumption, among 527 laryngeal cancer cases and 1297 controls. Italy and Switzerland, 1992–2000

gives the distribution of 527 laryngeal cancer cases and 1297 controls, and the corresponding ORs according to level of intake of fried foods. A significantly increased risk of laryngeal cancer was found for all the fried foods examined: the ORs for the highest level of consumption compared to the lowest one was 1.63 for beef or veal, 3.06 for fish or shellfish, 1.85 for eggs or omelette and 1.86 for potatoes. The trends in risk were highly significant for all the food items considered, with the exception of potatoes. Since a high intake of fried foods may simply be a marker of a low vegetable and fruit consumption – found to be strongly inversely related to laryngeal cancer risk in our dataset – the association of fried foods and laryngeal cancer was also evaluated after further adjustment for vegetable and fruit intake. The risk estimates were however not meaningfully modified. The associations between fried foods and laryngeal cancer risk was similar in various subsites, although higher ORs were observed for supraglottis than for glottis and others or unspecified sites.

Table 2

**Table 2:**
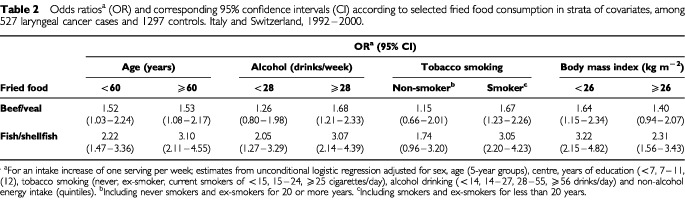
Odds ratios^a^ (OR) and corresponding 95% confidence intervals (CI) according to selected fried food consumption in strata of covariates, among 527 laryngeal cancer cases and 1297 controls. Italy and Switzerland, 1992–2000

shows the ORs for an increment of 1 weekly portion of fried meat or fish in strata of selected covariates. A consistent increased risk was observed in each stratum of age, alcohol drinking, tobacco smoking and body mass index. However, the association between the consumption of fried meat or fish was apparently stronger in older subjects (⩾60 years) in those with higher alcohol consumption (⩾28 drinks/week), in smokers or ex-smokers of less than 20 years, and subjects with a higher body mass index (⩾26 kg m^−2^).

## DISCUSSION

Our study suggests that consumption of fried foods is directly related to the risk of laryngeal cancer. A similar finding has been reported in another study which considered fried meat in relation to cancer of the larynx (Zheng *et al*, 1992), although there is evidence of a positive association between fried foods and cancer of the colorectum, stomach, as well as other neoplasms (IARC, 1993; Steineck *et al*, 1993). The associations between fried protein-rich foods and digestive tract cancers have been mainly attributed to the presence of genotoxic and mutagenic HA produced in cooked foods, particularly when a high temperature is reached, as in the case of frying (Övervik and Gustafsson, 1990; Layton *et al*, 1995; Skog *et al*, 1998; Sinha *et al*, 1998b). How these compounds could be involved in laryngeal carcinogenesis is less clear, although they may exert a particular effect on the tissues lining the upper aerodigestive tract, which enters in direct contact with them. Similar increased risks in relation to fried foods has also been observed in a companion case–control study of oral cancer (Franceschi *et al*, 1999), where the ORs for the highest level of consumption were 1.3 for meat, 1.2 for fish, 1.7 for eggs, and 1.4 for potatoes. Along the same line, there is the evidence of an excess lung cancer risk for exposure to HA from meats in rodents (Ohgaki *et al*, 1987) and humans (Sinha *et al*, 2000).

The role of HA could not explain the association found for fried potatoes, since they are produced essentially in the presence of protein. Fried potatoes, however, has been found to contain appreciable amounts of acrylamide, a chemical classified as a probable human carcinogen (IARC, 1994).

Factors other than the mutagenic activity of HA could also explain or confound the positive association of fried foods and laryngeal cancer. Meat and eggs themselves, irrespective of cooking methods, could be related to laryngeal cancer risk. In our data, however, the association of laryngeal cancer risk with meat or egg items consumed boiled was less strong than that with fried items, whereas no association was found for boiled fish or shellfish (Bosetti *et al*, 2002).

The role of fat for frying is a possible explanation of the increased risk observed. In particular mixed (unspecified) seed oils, frequently used for frying, have been shown in our dataset to be positively related to laryngeal cancer risk (Bosetti *et al*, 2002), and they could, at least in part, explain the positive association of fried foods. Furthermore, oils rich in n-6 polyunsaturated fats could be directly involved in the mutagenic activity of HA, enhancing their action through a promoting effect, more than other monounsaturated fats such as olive oil (Weisburger, 2000). However, the estimates for fried food consumption were only attenuated, but not materially modified, after further adjustment for consumption of mixed seed oils.

The hospital-based setting is a potential limitation of our study, since hospital controls may not be comparable with cases. However, controls were selected among patients with admission diagnosis not related to tobacco smoking, alcohol drinking and diet modifications. Moreover, in order to reduce any information bias, the questionnaire was administered to both cases and controls by the same interviewers and under similar conditions, and the food frequency intake for subjects interviewed at home was well comparable with that obtained at hospital (D'Avanzo *et al*, 1997).

The high participation rate of cases and controls, the comparable catchment areas of study subjects, the careful adjustment for tobacco and alcohol, as well as other potential confounding factors, are among the strengths of the study. Moreover, the use of FFQ, reported to be satisfactorily valid (Decarli *et al*, 1996) and reproducible (Franceschi *et al*, 1993), allowed to take into account the full dietary pattern, and to adjust for total energy intake (Willett and Stampfer, 1986).

In conclusion, our analysis suggests that the consumption of fried foods, irrespective of the content of foods, might be associated with an excess in laryngeal cancer risk. Selection, information bias or confounding are unlikely to explain this association, and its strength indicates that it is unlikely to be due to chance alone. In the absence of simple biological mechanisms, it is however unclear whether this implies causation, or a non-specific association with a poorer diet (La Vecchia *et al*, 1990).

## References

[bib1] ArcherVE1988 Cooking methods, carcinogens, and diet-cancer studies Nutr Cancer 11 7579 3283707 10.1080/01635588809513972

[bib2] BjeldandesLFMorrisMMFeltonJSHealySStuermerDBerryPTimourianHHatchFTHatchFT1982a Mutagens from the cooking of food. II. Survey by Ames' Salmonella test of mutagen formation in the major protein-rich foods of the American diet Food Chem Toxicol 20 357563 6751953 10.1016/s0278-6915(82)80099-9

[bib3] BjeldandesLFMorrisMMFeltonJSHealySStuermerDBerryPTimourianHHatchFTHatchFT1982b Mutagens from the cooking of food. III. Survey by Ames' Salmonella test of mutagen formation in the secondary sources of cooked dietary protein-rich Food Chem Toxicol 20 365369 6751954 10.1016/s0278-6915(82)80100-2

[bib4] BosettiCLa VecchiaCTalaminiRNegriELeviFDal MasoLFranceschiS2002 Food groups and laryngeal cancer risk: A case-control study from Italy and Switzerland Int J Cancer, 100 355360 12115553 10.1002/ijc.10485

[bib5] BreslowNEDayNE1980 Statistical methods in cancer research, Vol. 1 The analysis of case-control studiesIARC Sci Publ 32.Lyon: IARC 7216345

[bib6] D'AvanzoBLa VecchiaCKatsouyanniKNegriETrichopoulosD1997 An assessment, and reproducibility of food frequency data provided by hospital controls Eur J Cancer Prev 6 288293 9306076 10.1097/00008469-199706000-00006

[bib7] De StefaniERoncoAMendilaharsuMDeneo-PellegriniH1997a Case-control study on the role of heterocyclic amines in the etiology of upper aerodigestive cancers in Uruguay Nutr Cancer 32 4348 10.1080/01635589809514715 9824856

[bib8] De StefaniERoncoAMendilaharsuMGuidobonoMDeneo-PellegriniH1997b Meat intake, heterocyclic amines, and the risk of breast cancer. A case-control study in Uruguay Cancer Epidemiol Biomarkers Prev 6 573581 9264269

[bib9] DecarliAFranceschiSFerraroniMGnagnarellaPParpinelMTLa VecchiaCNegriESalviniSFalciniFGiacosaA1996 Validation of a food-frequency questionnaire to assess dietary intakes in cancer studies in Italy: results for specific nutrients Ann Epidemiol 6 110118 8775590 10.1016/1047-2797(95)00129-8

[bib10] DemirerTIcliFUzunalimogluOKucukO1990 Diet and stomach cancer incidence. A case-control study in Turkey Cancer 65 23442348 2346918 10.1002/1097-0142(19900515)65:10<2344::aid-cncr2820651030>3.0.co;2-h

[bib11] FranceschiSFaveroAContiETalaminiRVolpeRNegriEBarzanLLa VecchiaC1999 Food groups, oils and butter, and cancer of the oral cavity and pharynx Br J Cancer 80 614620 10408875 10.1038/sj.bjc.6690400 PMC2362347

[bib12] FranceschiSNegriESalviniSDecarliAFerraroniMFilibertiRGiacosaATalaminiRNanniOPanarelloGLa VecchiaC1993 Reproducibility of an Italian food frequency questionnaire for cancer studies: Results for specific food items Eur J Cancer 29A 22982305 8110502 10.1016/0959-8049(93)90225-5

[bib13] IARC, International Agency for Research on Cancer1993 Some naturally occurring substances: Food items and constituents, heterocyclic aromatic amines and mycotoxins IARC Monogr Eval Carcinog Risks HumVol. 56.Lyon, France: IARC

[bib14] IARC, International Agency for Research on Cancer1994 Some industrial chemicals IARC Monogr Eval Carcinog Risks HumVol. 60.Lyon, France: IARC

[bib15] KnektPSteineckGJarvinenRHakulinenTAromaaA1994 Intake of fried meat and risk of cancer: a follow-up study in Finland Int J Cancer 59 756760 7989114 10.1002/ijc.2910590608

[bib16] La VecchiaCNegriED'AvanzoBFranceschiSDecarliABoyleP1990 Dietary indicators of laryngeal cancer risk Cancer Res 50 44974500 2369728

[bib17] LaytonDWBogenKTKnizeMGHatchFTJohnsonVMFeltonJS1995 Cancer risk of heterocyclic amines in cooked foods: an analysis and implications for research Carcinogenesis 16 3952 7834804 10.1093/carcin/16.1.39

[bib18] NorellSEAhlbomAErwaldRJacobsonGLindberg-NavierIOlinRTornbergBWiechelKL1986 Diet and pancreatic cancer: a case-control study Am J Epidemiol 124 894902 3776972 10.1093/oxfordjournals.aje.a114479

[bib19] OhgakiHHasegawaHSuenagaMSatoSTakayamaSSugimuraT1987 Carcinogenicity in mice of a mutagenic compound, 2-amino-3,8-dimethylimidazo[4,5-f]quinoxaline (MeIQx) from cooked foods Carcinogenesis 8 665668 3581424 10.1093/carcin/8.5.665

[bib20] ÖvervikEGustafssonJ-Å1990 Cooked-food mutagens: current knowledge of formation and biological significance Mutagenesis 5 437446 2263202 10.1093/mutage/5.5.437

[bib21] PhillipsRL1975 Role of life-style and dietary habits in risk of cancer among Seventh-day Adventists Cancer Res 35 35133522 1192416

[bib22] SalviniSGnagnarellaPParpinelMTBoylePDecarliAFerraroniMGiacosaALa VecchiaCNegriEFranceschiS1996 The food composition database for an Italian food frequency questionnaire J Food Compos Anal 9 5771

[bib23] SinhaRKulldorffMCurtinJBrownCCAlavanjaMCRSwansonCA1998a Fried, well-done red meat and risk of lung cancer in women (United States) Cancer Causes Control 9 621630 10189048 10.1023/a:1008805525525

[bib24] SinhaRKulldorffMSwansonCACurtinJBrownsonRCAlavanjaMCR2000 Dietary heterocyclic amines and the risk of lung cancer among Missouri women Cancer Res 60 37533756 10919646

[bib25] SinhaRRothmanNSalmonCPKnizeMGBrownEDSwansonCARhodesDRossiSFeltonJSLevanderOA1998b Heterocyclic amine content in beef cooked by different methods to varying degrees of doneness and gravy made from meat drippings Food Chem Toxicol 36 279287 9651044 10.1016/s0278-6915(97)00162-2

[bib26] SkogKIJohanssonMAEJägerstadMI1998 Carcinogenic heterocyclic amines in model systems and cooked foods: a review on formation, occurrence, and intake Food Chem Toxicol 36 879896 9737435 10.1016/s0278-6915(98)00061-1

[bib27] SteineckGGerhardsson de VerdierMÖvervikE1993 The epidemiological evidence concerning intake of mutagenic activity from the fried surface and the risk of cancer cannot justify preventive measures Eur J Cancer Prev 2 293300 8358280 10.1097/00008469-199307000-00002

[bib28] SteineckGHagmanUGerhardssonMNorellSE1990 Vitamin A supplements, fried foods, fat and urothelial cancer. A case-referent study in Stockholm, 1985–87 Int J Cancer 45 10061011 2351481 10.1002/ijc.2910450604

[bib29] WardMHSinhaRHeinemanEFRothmanNMarkinRWeisenburgerDDCorreaPZahnSH1997 Risk of adenocarinoma of the stomach and esophagus with meat cooking method and doneness preference Int J Cancer 71 1419 9096659 10.1002/(sici)1097-0215(19970328)71:1<14::aid-ijc4>3.0.co;2-6

[bib30] WeisburgerJH2000 Approaches for chronic disease prevention based on current understanding of underlying mechanisms Am J Nutr 71 1710S1714S 10.1093/ajcn/71.6.1710S 10837325

[bib31] WillettWCStampferMJ1986 Total energy intake: implications for epidemiologic analyses Am J Epidemiol 124 1727 3521261 10.1093/oxfordjournals.aje.a114366

[bib32] World Cancer Research Fund in association with the American Institute for Cancer Research1997 Food, nutrition and the prevention of cancer: a global perspectiveWashington, DC: World Cancer Research Fund 10.1016/s0899-9007(99)00021-0 10378216

[bib33] ZhengWBlotWJShuX-OGaoY-TJiB-TZieglerRGFraumeniJrJF1992 Diet and other risk factors for laryngeal cancer in Shangai, China Am J Epidemiol 136 178191 1415140 10.1093/oxfordjournals.aje.a116484

